# Effects of Limited Wrist Motion and Forearm Rotation on Scapular Kinematics and Muscle Activity During Spoon-Feeding in Healthy Young Adults

**DOI:** 10.3390/jfmk11020135

**Published:** 2026-03-24

**Authors:** Noboru Chiba, Kazuki Ogawa, Ai Suzuki, Tadayoshi Minamisawa

**Affiliations:** 1 Department of Occupational Therapy, Yamagata Prefectural University of Health Sciences, Yamagata 990-2212, Japan; 2 Department of Convalescent Rehabilitation, Higashiosaka Hospital, Higashiosaka 536-0005, Japan; 3 Department of Rehabilitation, Tsuruoka Kyoritsu Hospital, Tsuruoka 927-8567, Japan; 4 Department of Physical Therapy, Yamagata Prefectural University of Health Sciences, Yamagata 990-2212, Japan

**Keywords:** feeding, inertial measurement units, electromyography, wrist orthosis

## Abstract

**Background:** Wrist–forearm orthoses used during self-feeding may alter scapular and shoulder mechanics and increase proximal load, but this has not been quantified. **Methods:** Seventeen right-hand-dominant young adults performed a spoon-feeding task under free and restricted conditions. A thermoplastic wrist–forearm orthosis positioned the wrist at approximately 30° dorsiflexion at rest and was intended to constrain wrist motion during the task without rigidly immobilizing forearm pronation–supination. Three-dimensional kinematics (scapula, shoulder, trunk, and distal joints) were recorded using inertial sensors, and surface electromyography was obtained from the upper trapezius, middle deltoid, and biceps brachii. Maximum joint angles and mean %MVC over the feeding cycle were compared between conditions (α = 0.05). **Results:** The restriction condition resulted in a more anteriorly tilted and downwardly rotated scapular posture, greater shoulder abduction and external rotation, and increased thoracic flexion, whereas maximum distal joint angles did not differ, suggesting a functional distal constraint rather than rigid immobilization. Middle deltoid and biceps brachii activities increased significantly, with a nonsignificant trend toward higher upper trapezius activation. **Conclusions:** In healthy young adults, limited wrist motion and forearm rotation during spoon-feeding were associated with altered proximal coordination, including scapular, shoulder/trunk, and proximal muscle changes.

## 1. Introduction

Eating and feeding are core activities of daily living (ADLs) that support health, participation, and independent living throughout the lifespan. The Occupational Therapy Practice Framework, 4th edition (OTPF-4), identifies feeding and eating as fundamental occupations within the scope of occupational therapy practice [1]. Occupational therapy practitioners routinely address feeding, eating, and swallowing by integrating biomechanical, environmental, cognitive, and psychosocial perspectives to promote the safe and effective performance of these activities [1,2]. Difficulties in self-feeding are strongly associated with reduced independence and poorer functional outcomes in populations with stroke and other neurological conditions, underscoring the importance of early and comprehensive interventions [2,3].

From a biomechanical standpoint, self-feeding is a complex multijoint task that requires coordinated movement of the trunk, shoulder girdle, elbow, forearm, wrist, and hand. Safaee-Rad et al. [4] reported that feeding with a spoon typically involves moderate shoulder abduction and rotational multi-joint with substantial elbow flexion, establishing functional ranges of motion for feeding activities. Subsequent three-dimensional (3D) motion analysis studies of drinking and eating tasks have further characterized joint contributions and movement phases, highlighting distinct kinematic patterns for reaching, scooping, transporting, and returning the hand to the starting position [5,6,7]. More recent studies using ecological self-feeding paradigms and instrumented utensils have confirmed that self-feeding kinematics can be captured reliably and that subtle changes in upper-limb motion are detectable in both healthy adults and clinical populations [3,8,9].

Upper-limb kinematic studies in people with stroke or hemiparesis have demonstrated that impairments often manifest as altered coordination between the shoulder and elbow joints during drinking or hand-to-mouth tasks, with increased shoulder abduction and compensatory trunk movements compared to healthy controls [3,8,9]. Phase-specific biomechanical analyses have also shown that proximal joint coordination during hand-to-mouth movements is sensitive to neuromuscular deficits and may provide clinically meaningful information for rehabilitation planning [6]. However, few studies have directly examined how an orthosis-based distal constraint, such as wrist immobilization with forearm extension that reduces distal degrees of freedom without assuming complete forearm fixation, affects proximal kinematics and muscle activity during self-feeding tasks in healthy adults. To our knowledge, no study has simultaneously quantified three-dimensional scapular kinematics, shoulder and trunk motion, and shoulder-girdle muscle activity during an ecologically valid spoon-feeding task while wearing a wrist–forearm orthosis that primarily restricts wrist motion in healthy adults.

The scapula plays a central role in coupling glenohumeral and thoracic motion and maintaining an efficient scapulohumeral rhythm. Previous three-dimensional studies have shown that scapular upward rotation, posterior tilt, and external rotation generally increase with humeral elevation [10,11]. Deviations from these patterns, such as decreased upward rotation or posterior tilt, are associated with subacromial pain, rotator cuff tendinopathy, and other shoulder disorders [12,13,14]. Experimental studies have also reported that task constraints, altered loading, or muscle fatigue can modify scapular motion, suggesting that the scapula dynamically adapts to changes in demand across the upper-limb kinetic chain [15,16]. For example, in individuals with massive rotator cuff tears, compensatory increases in scapular upward rotation during arm elevation have been interpreted as a strategy to offset reduced glenohumeral torque [17].

Movement restrictions extend beyond the wrist and forearm, manifesting in various clinical and experimental settings, such as orthotic use, joint immobilization, and neurological impairments [3,18,19,20,21,22,23]. These restrictions can alter the typical distribution of movement across upper-limb segments, prompting compensatory adjustments in proximal joints and muscles, along with the reorganization of task performance strategies [18,19,20,21,22,23]. The immobilization of the wrist, often achieved with forearm-based casts or splints, is common following injuries and surgeries, and patients frequently continue essential activities of daily living, such as self-feeding, while using these devices. Although immobilization safeguards tissues, it may shift task demands proximally and increase mechanical loading on the shoulder complex [18,19,20]. Experimental studies employing wrist orthoses during simulated work or functional tasks have demonstrated increased shoulder elevation and altered scapular muscle activation with restricted wrist motion [21,22,23]. However, while proximal compensation in response to distal restriction is generally acknowledged, quantitative evidence pertaining to specific functional activities remains limited. In particular, for spoon-feeding, a common activity of daily living demanding coordinated upper-limb control, the effects of a wrist-focused distal constraint using a wrist–forearm orthosis on scapular kinematics and shoulder girdle muscle activity have not been sufficiently clarified [8]. The present study contributes to the existing literature by quantifying compensatory responses during a specific functional activity, namely self-feeding. By simultaneously examining scapular kinematics, proximal upper-limb and trunk kinematics, and shoulder girdle muscle activity, it provides new insights into distal-to-proximal coordination during spoon-feeding.

Therefore, this study aimed to examine how a wrist–forearm orthosis providing wrist-focused motion restriction with limited forearm pronation–supination influences scapular kinematics, upper-limb joint angles, and shoulder girdle muscle activity during spoon-feeding in healthy young adults. The present study was designed to examine the acute effects of wrist-focused motion restriction during spoon-feeding, whereas longer-term adaptive responses were beyond its scope. Because compensatory responses to distal restriction may vary depending on task demands and individual movement strategies, the present hypotheses were intended to reflect one plausible pattern of proximal compensation during spoon-feeding. Based on previous findings on constrained and functional upper-limb tasks, we hypothesized that imposing a distal constraint (primarily at the wrist) would (1) increase shoulder abduction and elbow flexion, (2) maintain the scapula in a more anteriorly tilted and downwardly rotated posture, and (3) increase the activation of the upper trapezius, middle deltoid, and biceps brachii muscles. We further aimed to discuss how these biomechanical changes relate to occupational therapy approaches for clients with distal upper-limb impairments who struggle with self-feeding.

## 2. Materials and Methods

### 2.1. Study Design and Participants

This study employed a non-randomized, within-subject experimental design. All participants completed both conditions in a fixed order, with the free condition performed before the restriction condition. Seventeen right-hand-dominant university students (aged 21–22 years; height, 160.0 ± 3.0 cm; weight, 52.5 ± 6.5 kg) participated in this study. The inclusion criteria were as follows: (1) daily eating with the right hand; (2) no history of orthopedic or neurological disorders affecting the trunk or upper-limbs; (3) absence of current joint pain; and (4) no known allergy to dairy products (yogurt was used as the test food). Participants who experienced pain or discomfort during the practice trials were excluded.

All participants provided written informed consent. The study was approved by the Ethics Committee of Yamagata Prefectural University of Health Sciences (approval number: 2107-11) and conducted in accordance with the Declaration of Helsinki. Given its limited sample size and exploratory nature, this investigation should be considered a pilot study.

### 2.2. Instrumentation

#### 2.2.1. Equipment

**Figure 1:**
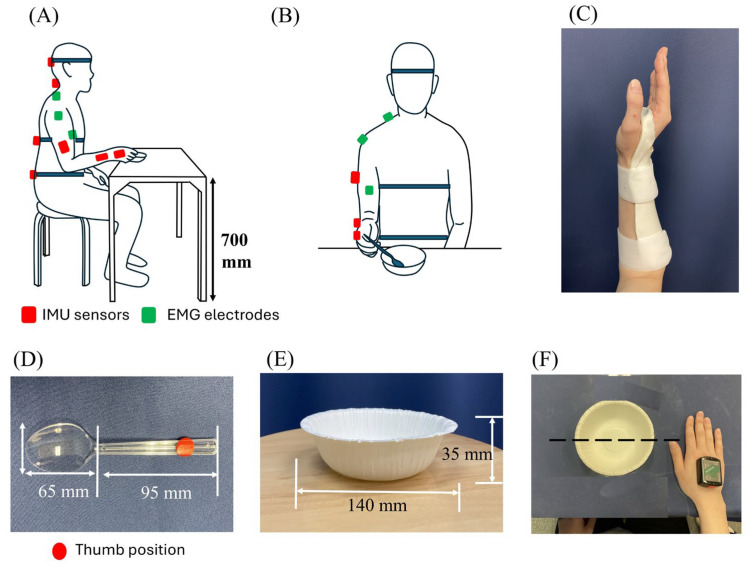
Experimental setup for the spoon-feeding task. (**A**) Side view of the participant and table showing the sitting posture and table height (700 mm). Inertia.l sensors (red markers) were attached to the trunk, head, and upper-limb segments, and the positions of surface electromyography electrodes are indicated in green. (**B**) Front view of the starting posture in the free condition. (**C**) Custom-made thermoplastic wrist–forearm orthosis used in the restriction condition, intended to constrain wrist motion with forearm-based fixation (the orthosis primarily constrained wrist motion and partially limited forearm pronation–supination, without assuming rigid immobilization). (**D**) Spoon used for the task, indicating bowl and handle lengths and the thumb reference mark. (**E**) Disposable paper bowl used for the task, indicating diameter and depth. (**F**) Top view illustrating the initial hand position relative to the paper bowl; the hand was positioned such that the tip of the thumb was aligned with the bowl’s centerline.

Upper-limb joint angles were recorded at 100 Hz using a wearable inertial motion analysis system (MyoMotion EM-M07; Noraxon, Scottsdale, AZ, USA) [24,25,26]. Each sensor (38 × 52 × 19 mm, 34 g) was mounted on eight body segments according to the Noraxon MR3.14 rigid body model: the external occipital ridge, C7, T12, sacrum (posterior), mid-right upper arm, mid-right forearm, dorsum of the right hand, and mid-right thigh (Figure 1). The segment local coordinate systems and anatomical axis conventions followed the vendor-defined MR3.14 model. Calibration was performed in a seated position with the elbow flexed at 90°. Wrist dorsiflexion and radial deviation were treated as positive values, whereas palmar (volar) flexion and ulnar deviation were treated as negative values. The sensors were attached using special fixation straps (for the pelvis and head) and an adhesive double-sided tape. The starting posture was as follows: the participants were seated in a chair with both hips and knees flexed at 90°, and holding the spoon with the forearm in a neutral pronation–supination position. A plastic spoon (bowl length: 65 mm, handle length: 95 mm) and a disposable paper bowl (diameter: 140 mm, depth: 35 mm) were used. The spoon was grasped at a standardized position with the thumb placed on a marked reference point on the handle, and the initial hand position was standardized by aligning the tip of the thumb with the paper bowl’s centerline (Figure 1E,F). This wearable inertial measurement unit(IMU)-based system has been widely used and has demonstrated good validity and reliability [26,27]. Two synchronized webcams (Logitech HD Webcam C920, Logitech, Lausanne, Switzerland) were positioned in the front and lateral directions to document task performance for subsequent verification. The videos were synchronized with the IMU system and were not used for kinematic estimation. To reduce potential magnetic disturbances and heading drift, the measurement area was kept free of ferromagnetic objects and electronic devices as much as possible, and the participants and sensors were consistently oriented across trials [28]. Calibration was repeated if abnormal heading behaviors (e.g., abrupt yaw changes) were observed during real-time monitoring. Prior to the experimental trials, a static stability check was performed in the same laboratory environment to quantify the drift over a duration comparable to the feeding task.

Surface electromyography (EMG) was performed using a wireless system (Clinical DTS; Noraxon Inc., Scottsdale, AZ, USA). Bipolar Ag/AgCl electrodes were placed over the muscle bellies of the right upper trapezius, middle deltoid, and biceps brachii after shaving and lightly abrading the skin, with an interelectrode distance of approximately 2 cm. These muscles were selected as representative proximal muscles because of their roles in scapular/shoulder control and elbow flexion during spoon-feeding. The EMG signals were processed using Noraxon MR3 (Noraxon U.S.A., Inc., Scottsdale, AZ, USA). The raw signals were band-pass filtered (FIR, 20–450 Hz; Hamming window, 49 points) to reduce movement artifacts and high-frequency noise, full-wave rectified, and low-pass filtered using a bidirectional IIR filter (Butterworth approximation, 20 Hz) to obtain a linear envelope. Maximum voluntary contractions (MVCs) for each muscle were obtained in standardized manual muscle testing positions, and EMG amplitudes during the feeding task were normalized to %MVC. The mean %MVC was calculated for each phase and for the entire feeding cycle for each muscle and condition.

Because a sternal sensor was not used, direct scapulothoracic (scapula-to-thorax) motion could not be quantified; thus, the scapular variables in this study should be interpreted as trunk-referenced, model-estimated scapular/shoulder-girdle orientation provided by the MR3.14 rigid-body model. To contextualize the potential trunk contributions, trunk kinematics were concurrently monitored using sensors placed at C7, T12, and the sacrum.

#### 2.2.2. Experimental Conditions and Orthosis

The participants performed the feeding task under two conditions:

1. Free condition: No external joint restriction, representing typical self-feeding behavior.
2. Restriction condition: A custom thermoplastic wrist–forearm orthosis was fabricated for each participant to restrict wrist motion while allowing functional grasping of the spoon. The orthosis extended to the forearm to enhance fixation and reduce excessive forearm rotation, although rigid immobilization of forearm pronation–supination was not intended.

The orthosis positioned the wrist at approximately 30° dorsiflexion at rest and was intended to constrain wrist motion during the task while allowing functional grasping of the object. The intended alignment and range of motion limits were visually confirmed using a goniometer before data collection. The orthosis was checked to ensure that it did not cause pain, numbness, or excessive pressure, and the participants practiced the task with and without the orthosis before recording. To reduce discomfort and allow acclimation to the orthosis, the participants were allowed to perform several brief practice movements after donning the orthosis before the recorded trials. The orthosis was intended to provide a functional distal constraint during spoon-feeding rather than rigid immobilization and was fitted to allow a comfortable grasp while avoiding excessive pressure on the hand. Nevertheless, forearm-based fixation was expected to partially limit pronation–supination and thereby reduce the distal degrees of freedom during the task. To minimize the potential carryover effects of the restriction condition on subsequent unrestricted performance, the free condition was always performed first, followed by the restriction condition [29,30].

We aimed to impose a distal constraint while preserving the feasibility of the task. Rigid immobilization of forearm rotation at a fixed angle would require an elbow-spanning design and could substantially hinder the feeding movements. Therefore, the proposed orthosis was designed to constrain wrist motion while partially limiting forearm pronation–supination without assuming rigid immobilization.

### 2.3. Task and Procedure

The participants sat on a chair with their hips flexed to approximately 90°, knees flexed to 90°, and feet flat on the floor. The table height was adjusted such that the elbows were flexed to approximately 90° in the initial posture. The paper bowl containing yogurt was placed at the sagittal midline of the body at a comfortable distance from the mouth. In the standardized starting position, the right upper arm was alongside the trunk, the elbow was flexed, and the spoon was held near the bowl with the forearm in a neutral position. The left hand was allowed to lightly stabilize the bowl but was not used to bring the bowl closer to the mouth.

Under each condition, participants were instructed to perform a self-paced feeding sequence similar to their usual eating speed.

1. Scooping: The act of scooping yogurt from a bowl using a spoon.
2. Transport to mouth: The spoon is brought to the mouth and yogurt is placed in the mouth.
3. Return: Return the spoon and upper-limb to the starting position, near the bowl.

The participants completed several practice trials under each condition to familiarize themselves with the tasks, orthoses, and equipment. The free condition was always performed first to capture each participant’s natural spoon-feeding pattern before exposure to the orthosis, thereby minimizing potential carryover of the restricted condition into subsequent unrestricted performance. Because the conditions were not counterbalanced, possible learning, adaptation, or sequencing effects cannot be fully excluded. During data collection, three trials were recorded for each condition, with short rest periods between the trials. Only the third trial was analyzed because the initial trials were more likely to reflect early familiarization with the task and orthosis, whereas the third trial was expected to better represent relatively stabilized performance under each condition [31]. Accordingly, the average of the three trials was not used, as it could have obscured the relatively stabilized performance intended for comparison.

### 2.4. Data Processing and Outcome Measures

Kinematic and EMG data were synchronized and exported for offline analyses. The feeding cycle was defined from the movement onset in the scooping phase to the completion of the return phase. Movement onset and transitions between the scooping, transport-to-mouth, and return phases were identified based on the joint angle time-series and confirmed by visual inspection of the measurement screen and video recordings when necessary. For graphical presentation, the joint angle trajectories were time-normalized to 0–100% of the feeding cycle, and vertical lines were used to indicate the boundaries between the three phases.

For each participant and condition, joint angles were computed according to the vendor-defined MR3.14 rigid-body model and expressed relative to the proximal segment or global/trunk reference as applicable (e.g., trunk and neck relative to the global reference; upper-limb segments relative to adjacent proximal segments). For the scapular variables, the angles represent model-estimated, trunk-referenced scapular/shoulder-girdle orientations derived from the IMU-based MR3.14 model, rather than direct measurements of scapulothoracic motion.

For EMG, the processed %MVC signals of the upper trapezius, middle deltoid, and biceps brachii were time-normalized to the feeding cycle, and the mean %MVC over the entire cycle was calculated for each muscle and condition. These three muscles were considered the predefined primary EMG outcomes. Scapular posterior tilt, downward rotation, shoulder abduction, and shoulder external rotation were predefined as primary kinematic outcomes based on the study aims; all other kinematic comparisons were considered exploratory.

### 2.5. Statistical Analysis

All statistical analyses were performed using R software (version 4.5.2; R Foundation for Statistical Computing, Vienna, Austria). For each joint angle variable, the maximum value observed during the feeding cycle was extracted for the free and restricted conditions. For electromyography, the mean %MVC over the entire feeding cycle was calculated for each muscle under each condition.

The normality of the paired data was assessed using the Shapiro–Wilk test applied to each condition and paired differences. When the distribution of the differences did not deviate significantly from normality, between-condition comparisons were conducted using paired *t*-tests. For variables that violated the normality assumption (biceps brachii activity), the Wilcoxon signed-rank test was used. An alpha level of 0.05 (two-tailed) was adopted for all the tests. The upper trapezius, middle deltoid, and biceps brachii muscles were designated a priori as the primary EMG outcomes. Secondary kinematic analyses were conducted on an exploratory basis. Because the study was designed as a pilot investigation with predefined primary outcomes and exploratory secondary kinematic analyses, formal correction for multiple comparisons was not applied to the secondary analyses to avoid an overly conservative interpretation in this preliminary setting. Accordingly, secondary kinematic findings were interpreted as exploratory.

Effect sizes for paired *t*-tests were expressed as *Cohen’s d*_z_ (mean difference divided by the standard deviation of the paired differences). For the Wilcoxon signed-rank tests, the effect size was expressed as the matched rank-biserial correlation. Ninety-five percent confidence intervals (95% CIs) were calculated for the mean paired differences (restriction − free) for normally distributed variables and for the Hodges–Lehmann estimate of the median paired difference for the biceps brachii. Descriptive statistics are presented as mean ± standard deviation (SD) for normally distributed variables and as median [interquartile range] for non-normally distributed variables.

## 3. Results

### 3.1. Scapular and Upper-Limb Joint Kinematics

Table 1 summarizes the maximum joint angles during the feeding task for each wrist condition. These scapular variables should be interpreted as model-estimated, trunk-referenced orientations rather than direct measurements of scapulothoracic motion. Compared with the free condition, the restriction condition resulted in significantly altered scapular and shoulder alignments. Scapular posterior tilt was smaller in the restriction condition (1° ± 7° vs. −4° ± 8°; *p* = 0.03, *Cohen’s d*_z_ = −0.56, 95% CI [−9.2, −0.4]), indicating a more anterior scapular posture in the restricted condition. Scapular downward rotation also increased in the restriction condition, as reflected by more negative values (−5° ± 3° vs. −8° ± 5°; *p* = 0.01, *Cohen’s d*_z_ = −0.70, 95% confidence interval [−5.9, −0.9]).

At the glenohumeral level, shoulder rotation (positive values indicated external rotation and negative values indicated internal rotation) shifted from a slightly internally rotated position in the unrestricted condition (−4° ± 14°) to a more externally rotated position in the restricted condition (13° ± 22°; *p* < 0.01, *Cohen’s d*_z_ = 0.79, 95% CI [5.8, 27.6]). Shoulder abduction also increased significantly under the restriction condition (53° ± 21° vs. 76° ± 22°; *p* < 0.01, *Cohen’s d*_z_ = 1.07, 95% CI [12.2, 34.7]). In addition, the participants adopted greater thoracic flexion when feeding with the orthosis (11° ± 6° vs. 18° ± 8°; *p* = 0.01, *Cohen’s d*_z_ = 0.93, 95% CI [3.2, 11.0]).

Time-series analysis using one-dimensional statistical parametric mapping (SPM1D) revealed phase-dependent kinematic deviations in the restriction condition compared to the free condition across multiple joints, providing a more granular view than the discrete maximum angles. In the distal segments, although the maximum wrist angles showed no statistical differences (Table 1), SPM1D identified significant clusters in wrist extension (Figure S2), whereas wrist radial/ulnar deviation showed limited or no significant supra-threshold clusters (Figure S1). These findings suggest that the orthosis constrained the dynamic movement trajectory across the feeding cycle, even when the participants reached similar peak angles at specific time points.

**Figure 2:**
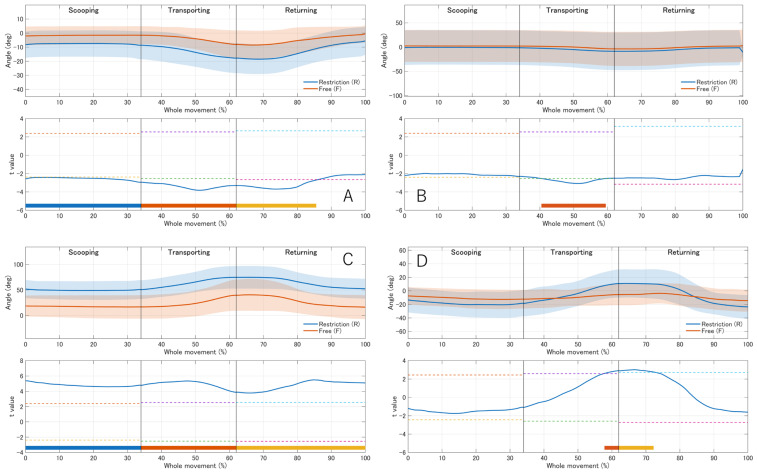
Comparison of scapular and shoulder kinematics during the spoon-feeding task (*n* = 17). Panels show (**A**) scapular posterior tilt, (**B**) scapular downward rotation, (**C**) shoulder abduction, and (**D**) shoulder external rotation. Upper panels show mean kinematic waveforms across the whole movement (0–100%) for the restriction (blue) and free (orange) conditions; shaded areas indicate ±1 SD. Vertical solid lines mark the phase boundaries (scooping, transporting, and returning) based on the mean phase ratios (Table S2). The lower panels display SPM{t} trajectories from paired *t*-tests computed separately for each phase and mapped onto the concatenated whole-movement timeline. Horizontal dashed lines indicate critical thresholds (α = 0.05, two-tailed). Colored bars indicate supra-threshold clusters (blue, scooping; red, transporting; orange, returning), representing significant differences within each phase.

Proximal compensatory movements were observed in response to these distal constraints. Specifically, the scapular posterior tilt was smaller (i.e., a more anteriorly tilted scapular posture) across substantial portions of the movement, and scapular downward rotation differed primarily during the transporting phase (Figure 2A,B; Table S1). Furthermore, shoulder abduction and external rotation were greater in the restricted condition, particularly as the spoon approached the mouth (Figure 2C,D). The trunk also contributed to compensation: thoracic flexion increased, with significant time-series differences observed in the thoracic region (Figure S6), whereas lumbar flexion did not show significant suprathreshold clusters (Figure S7; Table S1). Additional time-series results for scapular horizontal adduction are provided in Figure S8.

### 3.2. Muscle Activity

Table 2 presents the average muscle activity of the upper trapezius, middle deltoid, and biceps brachii during spoon-feeding under each condition. Upper trapezius activity tended to be higher in the restriction condition than in the free condition (23.5 ± 8.4% vs. 26.6 ± 8.6%); however, this difference was not statistically significant (*p* = 0.08, *Cohen’s d*_z_ = 0.45, 95% CI [−0.4, 6.8]).

In contrast, middle deltoid activity was significantly greater in the restriction condition (7.8 ± 4.2% vs. 10.7 ± 5.0%; *p* < 0.01, *Cohen’s d*_z_ = 1.28, 95% CI [1.7, 4.2]). The biceps brachii also showed a marked increase in activity with distal constraint (wrist-focused): median EMG rose from 5.4% MVC (interquartile range [IQR] 4.5–10.6) in the free condition to 10.8% MVC (IQR 9.0–12.1) in the restricted condition (*p* < 0.01, matched rank-biserial correlation = 0.94, Hodges–Lehmann median paired difference 5.0% MVC, 95% CI [1.4, 6.3]).

Taken together, these results demonstrate that the restriction condition required greater activation of the middle deltoid and biceps brachii, with a strong trend toward.increased upper trapezius activity, indicating higher proximal muscular demand during spoon-feeding when wrist motion was restricted.

### 3.3. Time-Series EMG Analysis

**Figure 3:**
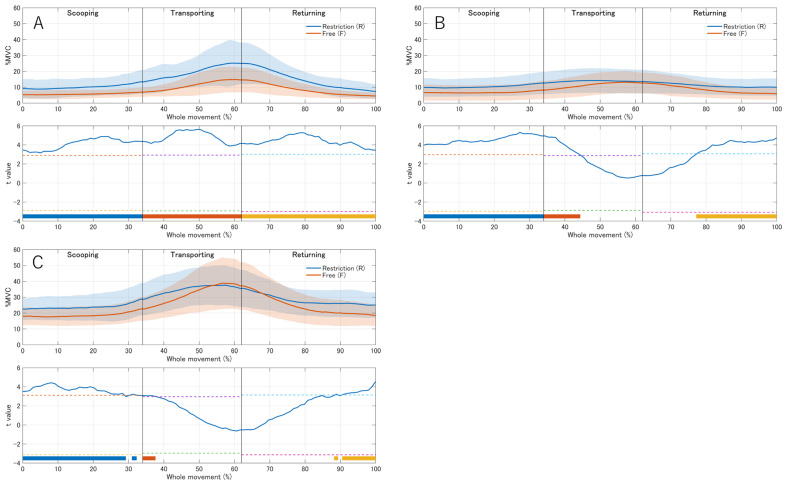
Concatenated time-series EMG activity and SPM1D results for (**A**) biceps brachii, (**B**) middle deltoid, and (**C**) upper trapezius (*n* = 17). Upper panels show the mean %MVC across the whole movement (0–100%) for the free (F) and restricted (R) conditions; shaded areas indicate ±1 SD. The lower panels show SPM{t} trajectories computed separately for each phase and mapped onto the concatenated whole-movement timeline. Colored bars indicate supra-threshold clusters (blue, scooping; red, transporting; orange, returning), representing significant differences within each phase. Vertical lines denote phase boundaries based on the mean phase ratios (Table S2).

To examine when condition-dependent changes occurred within the feeding cycle, the time-normalized %MVC waveforms were concatenated across the scooping, transporting, and returning phases using the mean phase ratios (Table S2) and compared between conditions using SPM1D paired *t*-tests (Table S3; Figure 3). For the biceps brachii, SPM1D revealed significantly greater activity in the restricted condition than in the free condition throughout all three phases, with the largest separation around the transporting-to-returning transition. For the middle deltoid, significant clusters were observed during the scooping phase, early portion of transport, and late portion of return, whereas no differences were detected during the remaining parts of transport and return. For the upper trapezius, significant clusters were identified mainly during the scooping and late return phases, with only limited significant intervals around the phase boundary during transportation. Overall, these time-series results indicate that wrist restriction consistently increased proximal muscle demand for the biceps brachii, whereas the deltoid and upper trapezius muscles showed phase-dependent increases.

## 4. Discussion

Previous studies have suggested that distal restriction can elicit proximal compensatory strategies; however, quantitative evidence during specific functional activities remains limited. The present study extends this knowledge by examining spoon-feeding, an ecologically relevant activity of daily living, under a feasible distal constraint involving limited wrist motion and forearm rotation. By simultaneously evaluating scapular kinematics, upper-limb joint motion, and shoulder girdle muscle activity, this study provides task-specific biomechanical evidence of how upper-limb coordination is reorganized during spoon-feeding under distal motion limitation.

The main findings were as follows: (1) distal constraint (primarily at the wrist) increased shoulder abduction and thoracic flexion, whereas distal joint angles remained largely unchanged; (2) the scapula was maintained in a more anteriorly tilted and downwardly rotated posture in the restriction condition; and (3) middle deltoid and biceps brachii activity increased significantly, with upper trapezius activity showing a non-significant trend toward higher values. Taken together, these results indicate that distal range-of-motion limitations are compensated for by more demanding proximal movement patterns during common ADLs. Beyond confirming a common clinical impression, the present study quantified the magnitude of these compensations and identified specific joints and muscles that are particularly affected during spoon-feeding with distal restriction.

From a clinical perspective, complete joint immobilization or ankylosis is relatively uncommon in self-feeding contexts, whereas many patients present with a restricted—but not absent—range of motion. Therefore, the present paradigm can be viewed as a clinically realistic distal-constraint model in which the wrist is effectively immobilized while the orthosis extends proximally to constrain distal motion without assuming the complete elimination of forearm pronation and supination.

### 4.1. Relationship with Previous Feeding and Kinematic Research

Our findings build on earlier studies describing upper-limb joint motion during feeding and drinking. Safaee-Rad et al. [4] reported characteristic patterns of shoulder abduction, internal rotation, and elbow flexion during feeding tasks and established reference ranges for the functional ROM. Subsequent 3D motion analysis studies of drinking from a glass and transporting a cup have demonstrated that healthy adults typically use coordinated shoulder elevation and elbow flexion with relatively modest trunk compensation [3,6,7]. In an ecological self-feeding paradigm, Nakatake et al. [8] showed that whole-body kinematics during eating can be decomposed into distinct phases, with shoulder abduction and elbow flexion peaking close to the mouth. More recent work using instrumented spoons and other sensor-based tools has confirmed that self-feeding kinematics can be captured reliably and that self-feeding is sensitive to upper-limb motor impairments [9]. Notably, the present participants were young, healthy adults, whereas previous studies examined older adults or people after stroke. Thus, the parallels in proximal kinematics should be interpreted as hypothesis-generating rather than direct evidence of clinical populations.

In contrast to these studies on unconstrained feeding tasks, our wrist-focused distal constraint model was intended to constrain wrist motion using a custom orthosis. Under these conditions, participants increased shoulder abduction and thoracic flexion to achieve the same functional goal of bringing food to the mouth, consistent with previous reports of compensatory proximal motion when distal function is compromised [18,19,20,23]. Notably, elbow flexion did not differ between conditions, suggesting that in this context, participants primarily relied on shoulder and trunk adjustments rather than additional elbow flexion to compensate for the distal constraint. The greater thoracic flexion observed in the constraint condition also aligns with the findings in stroke survivors and other neurological populations, who often adopt trunk movements to compensate for impaired upper-limb control during drinking and hand-to-mouth tasks [3]. Thus, even in healthy young adults, a relatively mild distal constraint is sufficient to elicit a pattern of proximal compensation similar to that observed in clinical populations. Notably, forearm supination did not differ in peak values between conditions (Table 1), whereas the time-series analysis revealed modest phase-specific differences (Figure S3).

### 4.2. Scapular Kinematics and Scapulohumeral Rhythm

Direct bone-pin measurements and 3D motion analysis have shown that scapular upward rotation, posterior tilt, and external rotation increase in a coordinated manner with humeral elevation in healthy individuals, reflecting an efficient scapulohumeral rhythm [10,11]. In the present study, the restricted condition was characterized by reduced posterior tilt and increased downward rotation compared with the free condition, a pattern that resembles scapular kinematics described in association with subacromial impingement and scapular dyskinesis [12,13,14]. Experimental studies manipulating task constraints or muscle fatigue have further demonstrated that scapular kinematics are sensitive to changes in loading and demands [15,16]. In individuals with massive rotator cuff tears, compensatory increases in scapular upward rotation during arm elevation have been interpreted as a strategy to offset reduced glenohumeral torque [17].

In the present study, participants in the restriction condition elevated their arms with greater shoulder abduction and maintained the scapula in a more anteriorly tilted and downwardly rotated position than those in the free condition, as indicated by the decreased posterior tilt and more negative downward rotation angles. Although these absolute differences in scapular angles were modest (approximately 5°), they represented a consistent shift toward a pattern associated with subacromial pain and rotator cuff pathology. These changes may be better interpreted primarily as biomechanical indicators of altered proximal coordination rather than direct evidence of clinical significance. However, because spoon-feeding requires coordinated multi-joint control, even relatively small scapular changes may reflect functionally relevant reorganization of upper-limb movement strategy. These small but systematic changes occurred alongside much larger increases in shoulder abduction and muscle activity, suggesting that even mild distal restriction can alter scapular/shoulder girdle orientation (trunk-referenced) as part of a global compensatory strategy. One possible interpretation is that the distal restriction altered the relative contribution of glenohumeral and scapular/shoulder-girdle motion patterns during feeding, leading participants to adopt a less optimal scapular orientation to preserve the hand trajectory and utensil orientation. Although our sample was young and asymptomatic, the repeated use of such compensatory patterns during daily self-feeding may warrant consideration of the cumulative loading of the shoulder complex, particularly in individuals with preexisting shoulder pathologies or limited muscular endurance.

Because a sternal sensor was not used, the scapular variables reported here reflect trunk-referenced, model-estimated scapular/shoulder-girdle orientation rather than direct scapulothoracic (scapula-to-thorax) motion and may incorporate concurrent trunk adjustments [32,33,34]. Notably, trunk angles (thoracic/lumbar/neck) were evaluated concurrently, allowing scapular changes to be interpreted in the context of a whole-body compensation strategy.

### 4.3. Muscle Activity and Kinetic Chain Considerations

The significantly higher activity of the middle deltoid and biceps brachii, together with the trend toward increased upper trapezius activation, indicates that distal limitation increases proximal muscular effort during spoon-feeding. Prior research has shown that scapular stabilizers and the deltoid are heavily recruited during arm elevation and that altered scapular kinematics are often accompanied by changes in the muscle activation patterns [13,16]. Studies on upper-limb tasks in people with neuromuscular or shoulder disorders have also reported that when distal or glenohumeral function is compromised, proximal segments adjust to preserve hand kinematics, often at the cost of increased muscle effort and fatigue [3,17].

Our results fit within this kinetic chain framework: the orthosis primarily targeted the wrist; however, phase-specific changes in forearm supination were also observed, suggesting task-level coupling and/or secondary constraint effects rather than rigid forearm immobilization. The associated increase in upper trapezius and middle deltoid activity likely reflects the need to stabilize and elevate the scapula and humerus to position the spoon appropriately. One possible interpretation is that the greater biceps brachii activity may have reflected stabilization and control of the spoon trajectory, as well as compensatory involvement in forearm rotational control. Although increased biceps brachii activity might be expected to accompany greater elbow flexion, the elbow angle did not differ between conditions. Because the biceps brachii contributes to both elbow flexion and forearm supination, increased activation under distal constraint may have been related to the reduced freedom of forearm rotation rather than to a larger elbow excursion. In a constrained multi-joint task, such as spoon-feeding, increased muscle activation does not necessarily translate into greater joint displacement because muscles may contribute to segmental stability and inter-joint coordination as well as to angular motion. Over time, such patterns could contribute to shoulder or neck discomfort in individuals who already have shoulder girdle or cervical spine vulnerability, especially when self-feeding is performed repeatedly while wearing a distal orthosis.

### 4.4. Implications for Occupational Therapy Practice

The OTPF-4 and related practice documents emphasize that occupational therapists address feeding, eating, and swallowing by considering the whole person and full task context rather than focusing solely on isolated joints [1]. The present findings reinforce the importance of this perspective. Where feasible, 3D motion analysis or simplified sensor-based tools, as described in recent occupational therapy and rehabilitation research, can provide objective insights into compensatory patterns [3,6,8,9]. For clients who demonstrate excessive shoulder abduction, scapular anterior tilt, or scapular elevation during feeding, interventions might include: (1) exercises targeting scapular stability and coordination; (2) task or environmental adaptations, such as adjusting table height, bowl position, or utensil design to reduce the need for extreme proximal motion; and (3) education on posture, pacing, and symptom monitoring to prevent overuse.

For occupational therapy education, this study can serve as an example of how distal impairments influence proximal movement during familiar ADLs. Incorporating simple kinematic observations or video-based analyses of self-feeding into laboratory classes may help students appreciate the interconnectedness of the upper-limb kinetic chain and recognize compensatory scapular strategies early in their training. Such learning experiences are consistent with calls within occupational therapy to integrate occupation-based tasks and biomechanical analyses into research and education [1,3].

### 4.5. Limitations and Future Research

This study has several limitations. First, the sample consisted of healthy young adults, which limits the generalizability of the findings to older adults and clinical populations. Because the sample consisted of young, healthy adults tested under experimental conditions, the present findings should not be directly extrapolated to patients who use splints for clinical indications.

Second, the task conditions were conducted in a fixed order (free followed by restriction), and three trials were recorded, but only the third trial was analyzed; therefore, potential sequence- or adaptation-related effects cannot be fully excluded. In addition, because a single trial rather than the average of multiple valid trials was used for analysis, within-subject reliability may have been reduced. The present findings should also be interpreted in light of the short-term experimental design. As the task was performed immediately after the orthosis was applied, the observed kinematic and EMG changes likely represent acute responses to an unfamiliar distal constraint. These initial compensations may differ from movement strategies adopted after repeated practice or prolonged orthosis use. Longitudinal studies are needed to determine whether the observed proximal compensations persist, diminish, or are reorganized over time.

Third, scapular variables were derived from skin-mounted inertial sensors without a sternal reference and should be interpreted as trunk-referenced, model-estimated shoulder-girdle orientations; thus, values are subject to soft tissue artifacts and may incorporate concurrent trunk adjustments [32,33,34]. In addition, although the orthosis provided functional distal constraint, the exact degree of motion restriction imposed during the task was not quantified continuously; therefore, the magnitude of the observed compensations should not be interpreted as a precise dose–response effect of distal restriction.

Finally, no a priori power analysis was performed, and multiple secondary variables were tested without formal multiplicity adjustment; therefore, findings for secondary outcomes should be interpreted as exploratory and confirmed in future studies.

Accordingly, the present findings should be interpreted as preliminary and hypothesis-generating rather than definitive, particularly considering the potential risk of insufficient statistical power.

## 5. Conclusions

In healthy young adults, distal motion limitation during spoon-feeding was associated with altered proximal coordination, including changes in scapular posture, shoulder/trunk kinematics, and proximal muscle activity. These findings provide preliminary biomechanical evidence of acute, task-specific compensatory responses rather than direct evidence of clinical significance. Given the small sample size and the use of a single functional task under experimental conditions, direct extrapolation to clinical populations should be conducted with caution. Further studies are warranted to determine whether similar patterns are observed across other activities of daily living and in patients who use splints for clinical indications.

## Figures and Tables

**Table 1:** Maximum joint angles during the feeding task under each wrist condition.

Variable	Free (°)	Restricted (°)	*p* Value	*Cohen’s d*_z_	95% CI
Scapular posterior tilt ★	1 ± 7	−4 ± 8	0.03	−0.56	[−9.2, −0.4]
Scapular downward rotation ★	−5 ± 3	−8 ± 5	0.01	−0.70	[−5.9, −0.9]
Shoulder external rotation ★	−4 ± 14	13 ± 22	<0.01	0.79	[5.8, 27.6]
Shoulder abduction ★	53 ± 21	76 ± 22	<0.01	1.07	[12.2, 34.7]
Thoracic flexion	11 ± 6	18 ± 8	0.01	0.93	[3.2, 11.0]
Neck flexion	12 ± 7	13 ± 8	0.47	0.17	[−2.3, 4.9]
Elbow flexion	130 ± 6	130 ± 6	0.99	0.00	[−4.2, 4.2]
Forearm supination	81 ± 16	78 ± 14	0.50	−0.17	[−11.1, 5.6]
Wrist extension	31 ± 11	34 ± 2	0.45	0.18	[−3.5, 7.7]
Wrist radial deviation	2 ± 6	2 ± 3	0.76	0.07	[−2.7, 3.6]

Values are mean ± SD. Effect sizes were reported as *Cohen’s d*_z_ for paired comparisons. Statistical significance was tested using a paired *t*-test (*p* < 0.05). ★ Predefined primary kinematic outcomes. 95% CI = 95% confidence interval for the mean difference (Restricted−Free).

**Table 2:** Muscle activity (%MVC) during spoon-feeding.

Variable	Free	Restricted	*p* Value	Effect Size	95% CI
Trapezius (upper)	23.5 ± 8.4	26.6 ± 8.6	0.08	0.45	[−0.4, 6.8]
Middle deltoid	7.8 ± 4.2	10.7 ± 5.0	<0.01	1.28	[1.7, 4.2]
Biceps brachii	5.4 [4.5, 10.6]	10.8 [9.0, 12.1]	<0.01	0.94	[1.4, 6.3]

Values are presented as mean ± SD, except for the biceps brachii, which is presented as median [interquartile range]. Effect sizes were reported as *Cohen’s d*_z_ for the upper trapezius and middle deltoid and as the matched rank-biserial correlation for the biceps brachii. The 95% CIs represent the confidence interval for the mean difference (Restricted−Free) for the upper trapezius and middle deltoid, and for the Hodges–Lehmann estimate of the median paired difference for the biceps brachii. Statistical significance was tested using paired *t*-tests (upper trapezius and middle deltoid muscles) and the Wilcoxon signed-rank test (biceps brachii muscle). Statistical significance was set at *p* < 0.05. CI = confidence interval.

## Data Availability

The data presented in this study are available from the corresponding author upon reasonable request. The data are not publicly available due to privacy and ethical restrictions.

## Supplementary Materials

The following supporting information can be downloaded at: https://www.mdpi.com/article/10.3390/jfmk11020135/s1. Table S1: Significant SPM1D clusters for joint-angle waveforms mapped onto the whole-movement timeline. Table S2: Mean phase proportions used for whole-movement concatenation and the number of significant SPM1D suprathreshold clusters for each muscle. Table S3: Significant SPM1D clusters (paired *t*-test) for EMG waveforms mapped onto the whole-movement timeline (0–100%). Figure S1: Concatenated time-series wrist radial/ulnar deviation angle and SPM1D results during spoon feeding. Figure S2: Concatenated time-series wrist extension angle and SPM1D results during spoon feeding. Figure S3: Concatenated time-series forearm supination angle and SPM1D results during spoon feeding. Figure S4: Concatenated time-series elbow flexion angle and SPM1D results during spoon feeding. Figure S5: Concatenated time-series neck flexion angle and SPM1D results during spoon feeding. Figure S6: Concatenated time-series thoracic flexion angle and SPM1D results during spoon feeding. Figure S7: Concatenated time-series lumbar flexion angle and SPM1D results during spoon feeding. Figure S8: Concatenated time-series scapular horizontal adduction angle and SPM1D results during spoon feeding.
