# Supplementary material for: Effects of Limited Wrist Motion and Forearm Rotation on Scapular Kinematics and Muscle Activity During Spoon-Feeding in Healthy Young Adults
Source: J Funct Morphol Kinesiol. 2026 Mar 24;11(2):135. doi: 10.3390/jfmk11020135 (PMC13108218; doi:10.3390/jfmk11020135)
Supplement: Supplementary file 1 [file jfmk-11-00135-s001.zip › Table S1.pdf]

Table S1. Significant SPM1D clusters for joint angle waveforms mapped onto the whole-movement timeline.

Variable (code)	No. of significant clusters	Cluster location on whole timeline (%)	Phase	Direction (R vs F)
**Elbow_Flex**	2	33.66–34.00	Scooping	R > F
		34.84–55.56	Transporting	R > F
**Forearm_Sup**	3	10.88–34.00	Scooping	R > F
		34.00–38.76	Transporting	R > F
		93.92–100.00	Returning	R > F
**Lumbar_Flex**	0	—	—	—
**Neck_Flex**	0	—	—	—
**Scap_DownRot**	1	40.44–59.48	Transporting	R < F
**Scap_Hadd**	0	—	—	—
**Scap_PostTilt**	3	0.00–34.00	Scooping	R < F
		34.00–62.00	Transporting	R < F
		62.00–85.56	Returning	R < F

Footnote: Significant SPM1D clusters (paired t-test) for joint angle waveforms mapped onto the concatenated whole-movement timeline (0–100%). The waveforms were concatenated using the group-mean phase proportions, and SPM1D paired t-tests were performed **within each phase**. The significant clusters were then mapped to the whole-movement axis.
