# Supplementary material for: Effects of Limited Wrist Motion and Forearm Rotation on Scapular Kinematics and Muscle Activity During Spoon-Feeding in Healthy Young Adults
Source: J Funct Morphol Kinesiol. 2026 Mar 24;11(2):135. doi: 10.3390/jfmk11020135 (PMC13108218; doi:10.3390/jfmk11020135)
Supplement: Supplementary file 1 [file jfmk-11-00135-s001.zip › jfmk-4195062-Figures S1-S8.pdf]

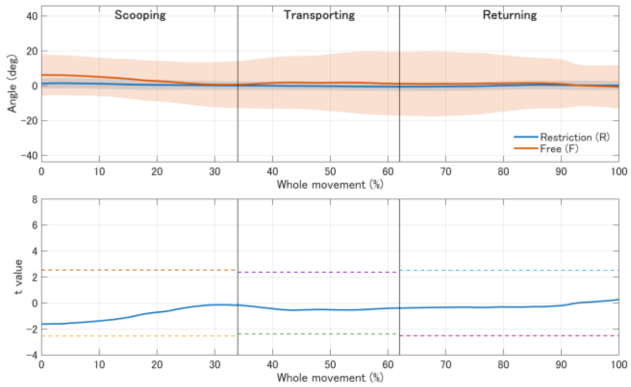

Figure S1. Concatenated time-series wrist radial/ulnar deviation angle and SPM1D results during spoon feeding.

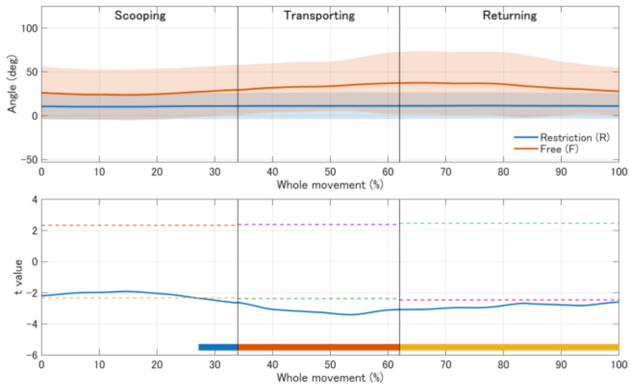

Figure S2. Concatenated time-series wrist extension angle and SPM1D results during spoon feeding.

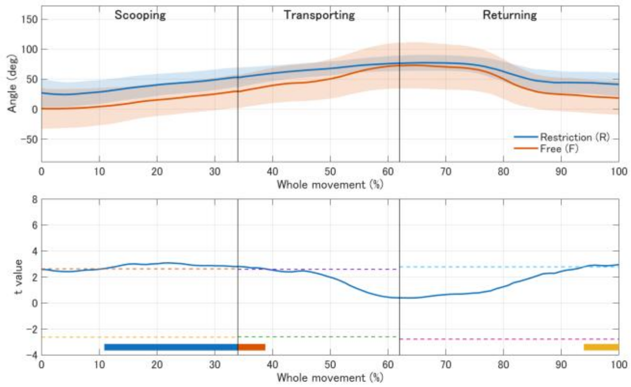

Figure S3. Concatenated time-series forearm supination angle and SPM1D results during spoon feeding.

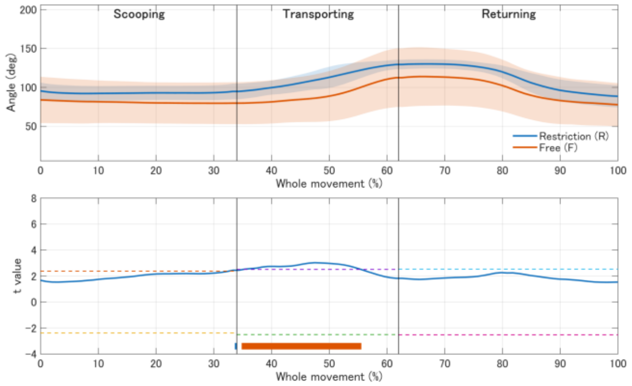

Figure S4. Concatenated time-series elbow flexion angle and SPM1D results during spoon feeding.

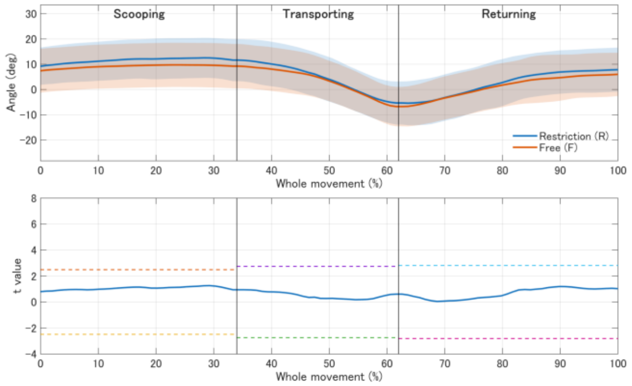

Figure S5. Concatenated time-series neck flexion angle and SPM1D results during spoon feeding.

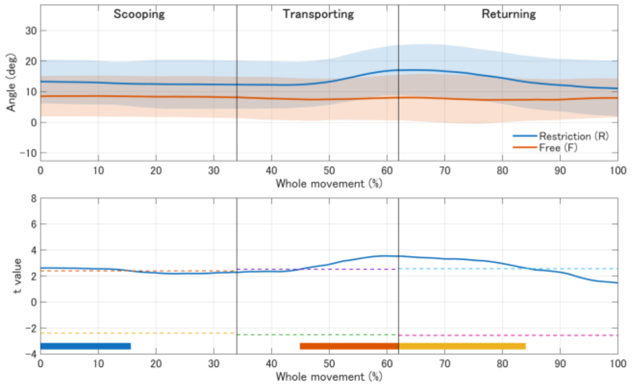

Figure S6. Concatenated time-series thoracic flexion angle and SPM1D results during spoon feeding.

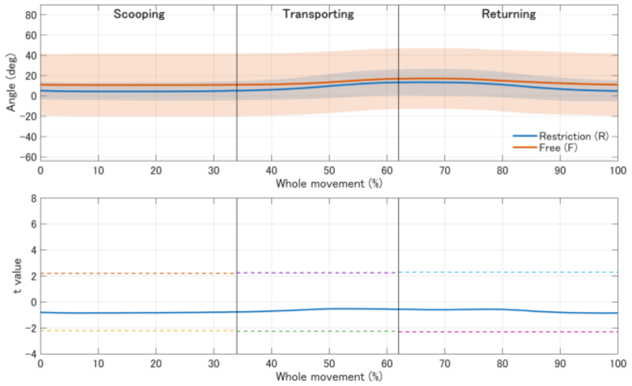

Figure S7. Concatenated time-series lumbar flexion angle and SPM1D results during spoon feeding.

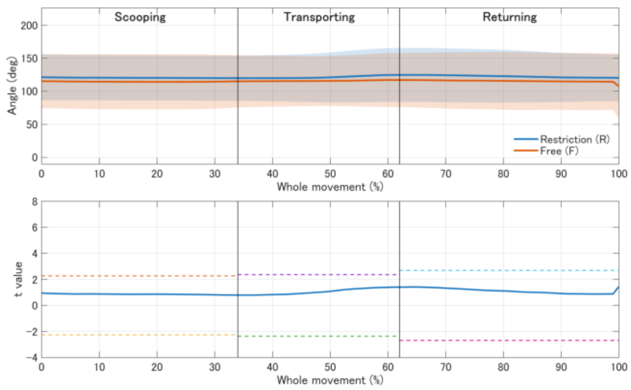

Figure S8. Concatenated time-series scapular horizontal adduction angle and SPM1D results during spoon feeding.
