# Supplementary material for: Effects of Limited Wrist Motion and Forearm Rotation on Scapular Kinematics and Muscle Activity During Spoon-Feeding in Healthy Young Adults
Source: J Funct Morphol Kinesiol. 2026 Mar 24;11(2):135. doi: 10.3390/jfmk11020135 (PMC13108218; doi:10.3390/jfmk11020135)
Supplement: Supplementary file 1 [file jfmk-11-00135-s001.zip › Table S2.pdf]

Table S2. Mean phase proportions used for whole-movement concatenation and number of significant SPM1D suprathreshold clusters for each muscle. The whole-movement timeline (0–100%) was constructed by concatenating the three phases according to group mean phase proportions. SPM1D paired t-tests were performed within each phase, and significant clusters were mapped onto the whole-movement timeline.

Muscle	Mean phase proportion (Scooping / Transporting / Returning)	Phase boundary on whole timeline (%) (S end ∣ T end)	No. of significant clusters (total)
UT	0.344 / 0.273 / 0.383	34.0 ∣ 62.0	5
MD	0.344 / 0.273 / 0.383	34.0∣ 62.0	3
BB	0.344 / 0.273 / 0.383	34.4 ∣ 61.9	3

Footnote: S, scooping; T, transporting; R, returning. The boundaries indicate the end of the scooping and transporting phases on the concatenated whole-movement axis.
