# Supplementary material for: Effects of Limited Wrist Motion and Forearm Rotation on Scapular Kinematics and Muscle Activity During Spoon-Feeding in Healthy Young Adults
Source: J Funct Morphol Kinesiol. 2026 Mar 24;11(2):135. doi: 10.3390/jfmk11020135 (PMC13108218; doi:10.3390/jfmk11020135)
Supplement: Supplementary file 1 [file jfmk-11-00135-s001.zip › Table S3.pdf]

Table S3. Significant SPM1D clusters (paired t-test) mapped onto the whole-movement timeline (0–100%). Positive clusters indicate higher values in the restriction condition than in the free condition (R > F).

Muscle	Cluster location on whole timeline (%)	Phase	Direction	Peak t (within cluster)
UT	0.00–29.24	Scooping	R > F	4.42
UT	30.94–32.30	Scooping	R > F	3.23
UT	34.00–37.64	Transporting	R > F	3.09
UT	88.22–89.36	Returning	R > F	3.22
UT	90.50–100.00	Returning	R > F	4.57
MD	0.00–34.00	Scooping	R > F	5.32
MD	34.00–44.36	Transporting	R > F	4.94
MD	77.20–100.00	Returning	R > F	4.73
BB	0.00–34.41	Scooping	R > F	4.90
BB	34.41–61.85	Transporting	R > F	5.64
BB	61.85–100.00	Returning	R > F	5.32

Footnote: Cluster ranges are expressed as percentages of the concatenated whole-movement axis (0–100%). “Direction” is based on the sign of the t-statistic (positive = R > F).
